# Implantable Polymer Scaffolds Loaded with Paclitaxel–Cyclodextrin Complexes for Post-Breast Cancer Tissue Reconstruction

**DOI:** 10.3390/polym17030402

**Published:** 2025-02-03

**Authors:** Liliana-Roxana Balahura (Stămat), Andreea Ioana Dinu, Adriana Lungu, Hildegard Herman, Cornel Balta, Anca Hermenean, Andreea Iren Șerban, Sorina Dinescu

**Affiliations:** 1 Department of Biochemistry and Molecular Biology, University of Bucharest, 050095 Bucharest, Romania; roxana.balahura@bio.unibuc.ro; 2 Advanced Polymer Materials Group, National University of Science and Technology Politehnica Bucharest, 011061 Bucharest, Romania; andreea_ioana.dinu@upb.ro (A.I.D.); adriana.lungu@upb.ro (A.L.); 3 “Aurel Ardelean” Institute of Life Sciences, Vasile Goldis Western University of Arad, 310414 Arad, Romania; hildegard.i.herman@gmail.com (H.H.); baltacornel@gmail.com (C.B.); anca.hermenean@gmail.com (A.H.); 4 Department Preclinical Sciences, Faculty of Veterinary Medicine, University of Agronomic Sciences and Veterinary Medicine of Bucharest, 050097 Bucharest, Romania; andreea-iren.serban@fmvb.usamv.ro; 5 Research Institute of the University of Bucharest, 050663 Bucharest, Romania

**Keywords:** triple-negative breast cancer, 3D scaffolds, paclitaxel, *NLRP3* inflammasome, pyroptosis

## Abstract

The side effects associated with the chemotherapy of triple-negative breast cancer (TNBC), such as nucleotide-binding oligomerization domain (NOD)-like receptor family (NLR), pyrin domain containing 3 (*NLRP3*) inflammasome activity, are responsible for the treatment failure and high mortality rates. Therefore, advanced delivery systems have been developed to improve the transport and targeted administration of anti-tumor agents at the tumor sites using tissue engineering approaches. Implantable delivery systems based on biodegradable polymers are an effective alternative due high biocompatibility, porosity, and mechanical strength. Moreover, the use of paclitaxel (PTX)-cyclodextrin complexes increases the solubility and permeability of PTX, enhancing the bioavailability and efficacy of the drug. All of these properties contribute to the efficient encapsulation and controlled release of drugs, preventing the damage of healthy tissues. In the current study, we detailed the synthesis process and evaluation of 3D scaffolds based on gelatin functionalized with methacryloyl groups (GelMA) and pectin loaded with PTX–cyclodextrin inclusion complexes on TNBC pathogenesis in vitro and in vivo. Bio-physio-chemical analysis of the proposed scaffolds revealed favorable mechanical and biological properties for the cellular component. To improve the drug solubility, a host–guest interaction was performed by the complexation of PTX with a cyclodextrin derivative prior to scaffold synthesis. The presence of PTX suppressed the growth of breast tumor cells and promoted *caspase-1* activity, the release of interleukin (IL)-1β, and the production of reactive oxygen species (ROS), conditioning the expression levels of the genes and proteins associated with breast tumorigenesis and *NLRP3* inflammasome. The in vivo experiments suggested the activation of pyroptosis tumor cell death, confirming the in vitro experiments. In conclusion, the bio-mechanical properties of the GelMA and pectin-based scaffolds as well as the addition of the PTX–cyclodextrin complexes allow for the targeted and efficient delivery of PTX, suppressing the viability of the breast tumor cells via pyroptosis cell death initiation.

## 1. Introduction

Triple-negative breast cancer (TNBC) is an aggressive neoplasm with an unpredictable evolution and limited therapeutic options, which is characterized by the absence of estrogen receptor, progesterone receptor, and human epidermal growth factor receptor 3 expression. Consequently, chemotherapy is the most widely used anti-tumor strategy for breast malignancies because targeted or hormone therapies are ineffective [1]. TNBC is associated with a poor prognosis due to the heterogenous nature of the tumor microenvironment (TME), which supports the epithelial-to-mesenchymal transition and metastasis mechanisms [2]. Moreover, breast TME is defined by a remarkable cellular diversity and interactions at the genetic and morphological levels and secreted molecules, all of them contributing to the growth of breast tumors [3]. Additionally, pro-inflammatory processes are present in the TME, orchestrating tumor development and progression through the secretion of chemokines, cytokines, or growth factors. One of the mechanisms associated with TNBC is the nucleotide-binding oligomerization domain (NOD)-like receptor family (NLR), pyrin domain containing 3 (*NLRP3*) inflammasome [4], which involves the recruitment of apoptosis-associated speck-like proteins containing a caspase-activation and recruitment domain (ASC), the cleavage and activation of *caspase-1*, and the maturation and secretion of interleukin (IL)-1β and IL-18. The activation of *caspase-1* promotes the cleavage of gasdermin D (GSDMD), whose amino-terminal fragment will contribute to the formation of GSDMD-derived pores and the secretion of IL-1β, ultimately determining the initiation of pyroptosis cell death [5]. Considering the consequences of *NLRP3* inflammasome signaling on TNBC pathogenesis, there is a strong need to understand their molecular mechanisms, in order to identify a potential effective therapeutic strategy.

Frequently, chemotherapy is initiated after the confirmation of oncological diagnosis, and numerous patients experience aggressive secondary effects. In some patients, surgical treatment (mastectomy or lumpectomy) is necessary, which will have a negative impact on patients both physically and psychologically. Therefore, targeted therapeutic approaches, which allow for the local administration of high concentrations of anti-tumor agents, are needed [6]. The systemic efficacy of anti-tumor agents, such as platinums or taxanes, is limited due to non-selective drug accumulation in non-target tissues, neurotoxicity, or the development of drug resistance [7]. To address these challenges, various scaffold-oriented approaches have been employed for precise drug delivery. Tissue engineering approaches aim to re-establish the normal functions of a damaged tissue by using tridimensional (3D), highly porous, and biodegradable scaffolds that support the cells’ adhesion, proliferation, migration, and differentiation. Different types of scaffolds have been designed to ensure the nutrients’ distribution and cell growth such as microspheres, hydrogels, fibers, composite polymer–bioceramic, or acellular scaffolds [8]. Natural polymer-based scaffolds facilitate biological processes by providing an optimal microenvironment for normal cellular behavior. Recently, scaffolds have been investigated for drug delivery applications in order to facilitate the administration of anti-tumor drugs to the tumor sites and decrease the cytotoxicity on non-target tissues [9]. Various biomaterial platforms are used as scaffolds for cancer therapy including nano-fiber membranes [10], films [11], or 3D printed structures [12]. Both the chemical composition of the scaffolds and their manufacturing strategies impact the physico-chemical properties and therapeutic activity of the materials [13,14,15,16]. Among all of the various drug delivery technologies envisioned for anti-tumor local treatments, hydrogels have drawn increasing recognition due to their composition and mechanical characteristics that could be adjusted to resemble biological tissues [17,18,19]. This advantage is further amplified by utilizing polymers sourced from natural origins to create biodegradable hydrophilic structures with outstanding biocompatibility and intricate porous formations without the need for solvents that could disrupt anti-tumor drug activity. This innovative approach not only minimizes the side effects commonly associated with chemotherapy, but also enhances the drug delivery precision, ensuring that treatments are effectively localized to target tumor sites [20,21,22] without the need for the scaffolds’ subsequent removal from the insertion site [23]. Such drug-loaded scaffolds, designed for local treatment, promote the precise delivery of drugs at the preselected tumor site, reducing systemic exposure [24] and drug hydrophobicity [25]. Amongst the most effective anti-tumor drugs of all time, paclitaxel (PTX) has been employed in treating lung, breast, and ovarian cancer, proving its ability to inhibit the growth of tumor cells. Recent studies have indicated the role of PTX as a microtubule stabilizing agent and as an anti-mitotic drug, which is used for tumor growth inhibition and the initiation of programmed cell death [26,27]. Moreover, the capacity of PTX to modulate the *NLRP3* inflammasome, in the TNBC context, has been investigated, and the obtained data highlighted that *NLRP3* pre-treatment facilitated the effects of PTX on tumor cells, therefore reducing the cancer-associated inflammation [28]. Nevertheless, PTX restricted water solubility poses a considerable drawback. To address this, different strategies have been exploited, generally using specific formulation vehicles such as Cremophor EL™, albumin, liposomes, micelles, or host–guest inclusion complexations [29]. Cyclodextrins exhibit a conical configuration, which integrates a hydrophilic exterior with a hydrophobic interior capable of encapsulating hydrophobic therapeutic molecules, such as PTX, the resultant inclusion complexes enhancing the water solubility of the drug [30,31].

Gelatin-derived composites have been recommended as the optimal media for the loading and release of various anti-tumor medicines [21]. Gelatin extracted from marine waste products, such as fish skin or bone, has been growing in popularity as a potential substitute for mammalian sources. This shift is attributed to its economically viable and environmentally friendly production methods as well as its low immunogenicity and reduced risk of disease transmission. Additionally, fish gelatin exhibits distinct physico-chemical properties including a low gelling point and low viscosity, facilitating the incorporation of components susceptible to heat due to its ease of dissolution and operating at room temperature [32,33,34].

The functionalization of gelatin with methacryloyl groups (GelMA) facilitates the obtaining of hydrogels through photopolymerization, a cell-friendly and biocompatible crosslinking strategy that expands the range of applications for targeted and controlled drug delivery. The physico-chemical and mechanical aspects of such scaffolds can be influenced through the GelMA synthesis conditions (bloom strength, functionalization degree), and/or crosslinking parameters (type and concentration of the used photoinitiator, curing time, polymer concentration) [35,36,37]. The successful encapsulation of PTX in GelMA-based hydrogels was reported by Vigata and co-workers, proving that the drug release kinetics are strongly subordinate on both the GelMA concentration and mechanical characteristics of the scaffolds [38]. Moreover, injectable microparticles synthetized using MA fish gelatin with encapsulated curcumin for gastric cancer treatment were recently reported by Zhu and coworkers [39].

To overcome the limitations of the constructs comprised solely of gelatin or GelMA, such as poor mechanical properties and fast degradation, various polysaccharides have been used to develop composite hydrogels as attractive platforms for drug solubilization and targeted delivery [40,41]. Furthermore, the interaction between polysaccharides and proteins can influence the protein functional capabilities, impacting some of the key drug-delivery system properties like swelling, biodegradability, and porosity [42]. Pectin, a plant polysaccharide exhibiting biological activity, possesses the ability to intervene in multiple stages of carcinogenesis such as the interaction with adhesion molecules, suppression of anti-apoptotic mechanisms, or tumor cell growth [43,44,45]. Moreover, pectin is considered as a promising candidate in this field due to its bioavailability, biocompatibility, muco-adhesive properties, and resistance to degradation. Depending on the degree of esterification, pectin can be classified into high-methoxyl esterified pectin (HP), low-methoxyl esterified pectin (LP), and low-methoxyl amidated pectin (AP) [46], with the chemical structure of pectin and the degree of esterification orchestrating its pharmacological activity.

Consequently, gelatin and pectin-based networks represent a highly promising combination for applications in cancer treatment and have been investigated regarding the delivery of anti-tumor agents; however, the impact of the specific type of pectin on the properties of the final material has not been considered. The synthesis of spherical medicated microglobules utilizing gelatin–pectin complex coacervations for drug administration has been previously documented, indicating significant promise for colon cancer therapy [47,48]. Additionally, nano-sized gelatin–pectin polyelectrolyte complexes containing curcumin have demonstrated exceptional safety and significant anti-tumor activity [49].

The remarkable tunable properties of protein–polysaccharide networks facilitate various drug-encapsulation methods including the integration of cyclodextrins via an acylation reaction. By forming host–guest inclusion complexes with PTX, the solubility of PTX into the hydrogels is increased, creating thus a complex delivery system for PTX for breast cancer therapy [50,51]. However, to the extent of our knowledge, hydrogel formulations based on GelMA and pectin that encapsulate host–guest inclusion complexes based on cyclodextrin and PTX for local breast cancer treatment have yet to be studied.

The present study represents a complex approach that encompasses both tissue engineering and drug delivery directions that considered two main objectives: (1) the synthesis and investigation of novel 3D scaffolds composed of GelMA and two distinct pectins (AP or HP) in order to create biocompatible and biodegradable scaffolds with optimal physico-chemical properties for cell growth, and (2) the evaluation of PTX–cyclodextrin complexes embedded into GelMA and pectin-based scaffolds to promote the pyroptosis initiation of the tumor cells by enhancing the *NLRP3* inflammasome activity both in vitro and in vivo. A dual photo-enzymatic crosslinking approach (photochemical crosslinking succeeded by enzymatic crosslinking using transglutaminases) was utilized to establish covalent bonds between the pectin and GelMA macromolecules, therefore forming a hydrophilic polymeric matrix designed to serve as drug-delivery systems for PTX. In vitro experimental conditions provide a controlled microenvironment for scientific studies, with numerous advantages such as low costs, high stability, and reproducibility. However, in vivo studies are necessary to confirm the in vitro results since the biological processes from a living organism are difficult to reproduce due to the complexity of cellular behavior and interconnected molecular pathways [52]. Therefore, the aim of this study was to obtain a biocompatible 3D porous biosystem enriched with an anti-tumor component designed to inhibit breast tumor cells and induce pyroptosis by activating the *NLRP3* inflammasome.

## 2. Materials and Methods

### 2.1. Materials

Gelatin from fish skin (Sigma-Aldrich-Gelatin from cold water fish skin, BioReagent, Steinheim, Germany) was methacrylated, according to the protocol described in a previous report [53], to a methacrylation degree of 33%. High-methoxyl pectin (HP) with 70–75% esterification was acquired from Sigma-Aldrich, Steinheim, Germany. A food-grade amidated pectin (Grinsted^®^ LA 410) derived from citrus peel (Danisco, Czech Republic), with a 29–33% degree of esterification and 17–21% degree of amidation (as specified by the provider), was kindly donated by KUK Romania and utilized without additional purification. Food-grade microbial transglutaminase with an enzyme activity of 80–120 U/g was provided by RAPS Romania and utilized as received. PTX was supplied by Acros Organics, ThermoFisher Scientific, Waltham, MA, USA, while methacrylic anhydride, Irgacure 2959 and hydroxypropyl-β-cyclodextrin (HP-β-CD) were procured from Sigma-Aldrich, Steinheim, Germany and used as received. Phosphate buffer saline (PBS) tablets producing 10 mM phosphate, 2.7 mM KCl, and 0.14 M NaCl, pH 7.4, at 25 °C were acquired from Carl Roth, Karlsruhe, Germany.

Cell lines used in this study (MCF-12A and MDA-MB-231) were obtained from ATCC, Manassas, USA and maintained in culture according to the instructions, while the necessary media (Dulbecco’s modified Eagle’s medium (DMEM), Dulbecco’s modified Eagle’s medium/Nutrient Mixture F-12 Ham (DMEM-F12), supplements (antibiotic antimycotic solution, PBS powder, Cholera toxin from *Vibrio cholerae* supplement, human epidermal growth factor (hEGF) supplement, hydrocortisone supplement) were purchased from Sigma-Aldrich, Steinheim, Germany, and fetal bovine serum (FBS) was obtained from ThermoFisher Scientific, Waltham, MA, USA.

For the biocompatibility assays, we used thiazolyl blue tetrazolium bromide (MTT) powder and an In Vitro Toxicology Assay Kit purchased from Sigma-Aldrich, Steinheim, Germany, while a LIVE/DEAD Viability/Cytotoxicity Kit for mammalian cells was purchased from ThermoFisher Scientific, Waltham, MA, USA. The Caspase-Glo 1 Inflammasome Assay and Lumit Human IL-1β Immunoassay were purchased from Promega, Madison, WI, USA. Amplex Red Hydrogen Peroxide/Peroxidase, the WesternBreeze Chromogenic Kit, anti-mouse and anti-rabbit, and SeeBlue Plus2 Pre-stained Protein Standard were purchased from ThermoFisher Scientific, Waltham, MA, USA. Methanol was purchased from Scharlau, Barcelona, Spain. Bovine serum albumine (BSA), triton-X100, paraformaldehyde (PFA) solution, Hoechst 33,258 solution, Trizma hydrochloride, Bradford reagent, 2-mercaptoethanol, acrylamide/bis-acrylamide 30% solution, ammonium persulfate, Trizma base, and sodium deoxycholate (SDS) were purchased from Sigma-Aldrich, Steinheim, Germany. The Immobilon-P polyvinylidene fluoride (PVDF) membrane was purchased from Merck, Darmstadt, Germany. Glycine and N,N,N’,N’-tetra-methylethylenediamine were purchased from BioRad, Hercules, CA, USA. *Caspase-1* antibody was purchased from Cell Signaling Technology, Inc., Danvers, MA, USA. P53, *BRCA1*, and *NLRP3* antibodies were purchased from SantaCruz Biotechnology, Inc., USA. The *GAPDH* antibody was purchased from Proteintech, Planegg-Martinsried, Germany. Goat anti-mouse AlexaFluor 488, goat anti-mouse AlexaFluor 546, and goat anti-rabbit AlexaFluor 647 secondary antibodies were purchased from Invitrogen, Waltham, MA, USA. For molecular analysis, we used the TRIzol reagent purchased from Sigma-Aldrich, Steinheim, Germany and the iScript cDNA Synthesis Kit purchased from BioRad, Hercules, CA, USA. The Forget-Me-Not Evagreen qPCR Master Mix was purchased from Biotium, Fremont, CA, USA.

### 2.2. Preparation and Characterization of PTX:Cyclodextrin Inclusion Complexes

An HP-β-CD derivative was used as the complexing agent for PTX, and the PTX:HP-β-CD inclusion complexes were prepared at a stoichiometric molar ratio of 1:20 via an aqueous solution-stirring method and then dehydrated through lyophilization [54]. An excess amount of HP-β-CD was used in order to increase the complexation yield. The resultant white powder was suspended in distilled water, vortexed, and centrifuged. The supernatant was collected and filtered, representing the PTX:HP-β-CD inclusion complexes, while the precipitate was used as a reference system consisting of a physical mixture of the two components.

A comprehensive characterization study of the PTX:HP-β-CD inclusion complexes was performed using Fourier transform infrared spectroscopy (FTIR), differential scanning calorimetry (DSC), and thermogravimetric analysis (TGA).

A Vertex 70 Bruker FTIR set up in attenuated total reflectance (ATR) mode was used to record the FTIR spectra of the pure PTX, HP-β-CD, their physical mixture, and their inclusion complex, respectively. The study was conducted at a resolution of 4 cm^−1^ including 32 scans across the range of 600 to 4000 cm^−1^ at ambient temperature.

To study the interplay between PTX and the HP-β-CD derivative, advanced thermoanalytical investigations were performed using a Netzsch STA 409 PC Luxx Simultaneous Thermal Analyzer, which operates both thermogravimetric and calorimetric analyses. Aluminum plates holding about 5 mg of sample were heated from 25 to 300 °C at a rate of 5 K/min under a nitrogen purge.

### 2.3. Preparation of Drug-Loaded Scaffolds

**Figure 1:**
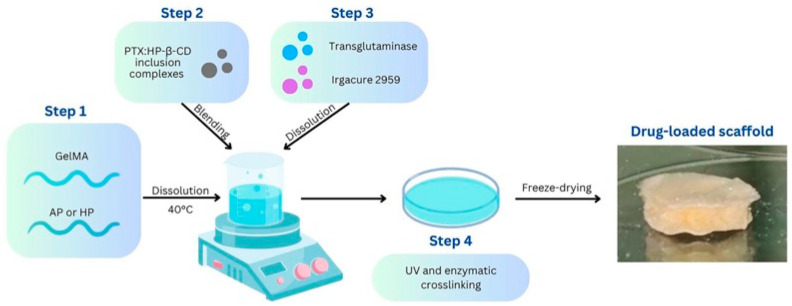
Schematic depiction of the drug-loaded hydrogel scaffolds.

Formulations containing the PTX:HP-β-CD inclusion complexes were prepared using a combination of a methacryloyl derivative of gelatin (GelMA) and pectin (AP or HP), as graphically depicted in Figure 1. For this purpose, GelMA with a substitution degree of 33% was synthesized by chemically altering the gelatin using methacrylic anhydride [34]. The polymeric mixtures were synthetized by dissolving GelMA (10% wt/wt) and either AP or HP (2.5% wt/vol) in distilled water under magnetic stirring at 40 °C. A precise amount of PTX:HP-β-CD (2% wt/vol) was then added to the polymeric mixtures and the samples coded as GFAP + PTX and GFHP + PTX, respectively, were obtained. To investigate the influence of pectin type (amidated or non-amidated) on the material composition, samples containing solely GelMA and PTX:HP-β-CD inclusion complexes were also prepared and labeled as GF + PTX. Following the same protocol, samples without the PTX:HP-β-CD inclusion complexes were also prepared and used as reference materials (denoted as GF, GFAP, GFHP). Transglutaminase and Irgacure 2959 were included into each mixture at doses of 5 U/mL and 0.1% wt/wt, respectively, to facilitate both enzymatic (24 h, 37 °C) and UV light-induced photo-crosslinking (365 nm, 5 min each side). All the obtained samples were meticulously washed using distilled water to eliminate any unincorporated or loosely adhered chemicals and subsequently freeze-dried.

### 2.4. Preliminary Characterization of Drug-Loaded Scaffolds

Composition-dependent swelling behavior and morphological properties of the obtained hydrogels were further evaluated.

To assess the swelling behavior, samples of each composition were precisely weighed to determine the dry weight (w_d_) and further submerged in PBS at room temperature. The wet weight (w_w_) of the specimens was periodically assessed by extracting the samples from the PBS, lightly blotting them using filter paper, and promptly weighing them at regular time intervals for up to 6 h. The swelling degree including the maximum swelling degree (MSD) was calculated using Equation (1).

(1) $$MSD=\frac{w_{w}-w_{d}}{w_{d}}$$

The internal microstructure of the scaffolds was examined with micro-computed tomography (μ-CT) utilizing a SkyScan 1272 high-resolution device. Each set of data was acquired by scanning the lyophilized samples, which were secured to a holder using modeling paste, positioned in front of a 50 kV voltage source and 200 μA current, during the 180 revolution with an increment of 0.2°. 3D depictions were captured with an image pixel size (scanning resolution) of 1.5 μm. Subsequently, the data were analyzed with the CT NRecon program (Bruker) and rebuilt into micrographs of three-dimensional objects with CT-Vox.

### 2.5. In Vitro Experiments

#### 2.5.1. Cell Culture and Subcultivation

Two cell lines were used for this experimental design: the MCF-12A normal breast cell line and MDA-MB-231 tumor cell line. The MCF-12A cell line was used to gain insights into the effect of the original proposed scaffolds in non-pathological conditions due to its non-tumorigenic potential and the expression of characteristic epithelial and mesenchymal markers. Moreover, MCF-12A cells represent an optimal experimental model due to the epithelial origin of the cells, which is suitable for the study of the *NLRP3* inflammasome [55]. On the other hand, the MDA-MB-231 cell line is specific for the study of TNBC and the epithelial–mesenchymal transition mechanisms due to the aggressive origin and high metastatic potential of tumor cells [56].

The culture media for the MCF-12A cells was represented by a mix consisting of DMEM-F12 media supplemented with 10% FBS, 1% antibiotic-antimycotic solution, 100 ng/mL Cholera toxin from *Vibrio cholerae* supplement, 20 ng/mL hEGF supplement, and 500 ng/mL hydrocortisone supplement, while a simplified formula consisting of DMEM supplemented with 10% FBS and 1% antibiotic-antimycotic was used to maintain the MDA-MB-231 cells in culture in standard conditions.

#### 2.5.2. Achievement of 3D Biosystems

The 1 cm^2^ diameter samples from each proposed GF-derived scaffold were sterilized using UV light. MCF-12A cells were seeded in the 3D scaffolds at a 2.5 × 10^5^ cells/cm^2^ density, while the MDA-MB-231 cells were seeded in the 3D scaffolds at a 2 × 10^5^ cells/cm^2^ density and allowed to adhere to the porous structure of the 3D scaffolds. The biocompatibility assessment was accomplished at 3 and 7 days post-cell seeding, and *caspase-1* activity, IL-1β release, and H_2_O_2_ production were performed at 12 h and 24 h post-cell seeding.

#### 2.5.3. In Vitro Biocompatibility Evaluation of 3D Biosystems

In order observe the biocompatibility of the GF-based scaffolds, the breast cells’ viability and proliferation and the scaffolds’ cytotoxicity were evaluated at 3 and 7 days after cell-seeding by employing the MTT, LDH, and LIVE/DEAD assays.

The MTT assay was performed to observe the viability and proliferation capacity of the cells in contact with the proposed GF-based scaffolds. The obtained 3D biosystems were mixed with 1 mg/mL MTT for 4 h in standard conditions. After the incubation period, the formazan crystals formed by the metabolic active cells were solubilized with isopropanol, and the absorbance was measured at 550 nm using a FlexStation 3 Spectrophotometer (Molecular Devices, San Jose, CA, USA).

The GF-based scaffolds’ cytotoxicity upon the cells was quantified based on the amount of lactate dehydrogenase (LDH) enzyme released by the damaged cells. The conditioned culture media were collected and incubated with LDH assay-specific reagent, following the manufacturer’s instructions. The absorbance was measured at 490 nm using a FlexStation 3 Spectrophotometer.

LIVE/DEAD fluorescence staining coupled with confocal microscopy was used in order to evaluate the status of the cell culture. The staining solution contains two fluorescence dyes, calcein acetomethoxy (AM), which allows for the observation of live cells in a green fluorescence, and ethidium bromide homodimer (EtBr), which allows for the observation of the dead cells’ nuclei in a red fluorescence. The obtained 3D biosystems were mixed with the staining reagent following the manufacturer’s instructions. The visualization and image acquisition was performed using the Nikon A1/A1R Confocal Laser Microscope System (Melville, NY, USA). The quantification of fluorescence levels was performed using ImageJ software (version 1.x bundled with 64-bit Java 8).

#### 2.5.4. *Caspase-1* Activity—Caspase-Glo 1 Inflammasome Assay

*Caspase-1* activity was monitored and determined using the Caspase-Glo 1 Inflammasome Assay. This bioluminescent method combines the Z-WEHD-aminoluciferin substrate cleavage by active *caspase-1* and the generation of light by recombinant luciferase. To determine the *caspase-1* activity, culture media from the obtained 3D biosystems were collected and incubated with the kit’s reagents, according to the recommended protocol. The luminescent signal was measured using FlexStation 3.

#### 2.5.5. IL-1β Release—Lumit Human IL-1β Immunoassay

The IL-1β levels from the proposed 3D biosystem were determined using the Lumit Human IL-1β Immunoassay, according to the manufacturer’s indications. The culture media were collected from each 3D biosystem and incubated with the corresponding reagents. The amount of cytokine released into culture media was correlated with the luminescent signal registered. Quantification of the IL-1β levels was realized using a 40 ng/mL–22 pg/mL range standard curve. The luminescent signal was measured at 12 h and 24 h using FlexStation 3.

#### 2.5.6. ROS Generation—Amplex Red Hydrogen Peroxide/Peroxidase Assay

The levels of H_2_O_2_ produced by the cells embedded into GF-based scaffolds were measured using the Amplex Red Hydrogen Peroxide/Peroxidase Assay. In the presence of peroxidase, the Amplex Red reagent (10-acetyl-3,7-dihydroxyphenoxazine) reacts stoichiometrically with H_2_O_2_, resulting in a fluorescent red oxidation product named resorufin. The experimental protocol was carried out according to the manufacturer’s instructions. The formation of resorufin was analyzed at 550 nm excitation and 590 nm emission, respectively, employing FlexStation 3.

#### 2.5.7. Gene Expression—Quantitative Real-Time PCR

The gene expression evaluation was performed by quantitative Real-Time PCR (qRT-PCR), considering a comparison between the normal MCF-12A cells and the tumor MDA-MB-231 cells exposed to GF-based scaffolds. The total RNA was isolated using the classic method with TRIzol reagent. The concentration and purity of the obtained RNA samples were measured using a NanoDrop 8000 spectrophotometer (Thermo Scientific, Waltham, MA, USA). The complementary DNA (cDNA) was obtained using the iScript cDNA Synthesis Kit and the Veriti 96-Well Thermal Cycler (Applied Biosystems, Waltham, MA, USA). Gene expression analyses were performed using a ViiA 7 Real-Time PCR System (Applied Biosystems, Waltham, MA, USA), Forget-Me-Not EvaGreen qPCR Master Mix, and specific primer sequences (Table 1). All of the samples were processed in triplicate, and the expression of glycerol aldehyde phosphate dehydrogenase (*GAPDH*) was used as the reference. The expression levels were determined using the 2^−ΔΔC(t)^ method, and the data were normalized to the control.

#### 2.5.8. Protein Expression—Immunostaining Coupled with Confocal Microscopy

Fluorescent staining coupled with confocal microscopy allowed for the visualization of the protein of interest. A 4% PFA solution was used to fix the 3D biosystems and were later were permeabilized using 0.1% Triton-X100 solution in 2% BSA for 30 min at 4 °C. The 3D biosystems were incubated overnight at 4 °C with the p53 mouse monoclonal antibody (1:100), *BRCA1* mouse monoclonal antibody (1:100), *NLRP3* mouse monoclonal antibody (1:100), and *caspase-1* rabbit polyclonal antibody (1:200) and then with the goat anti-mouse secondary antibody AlexaFluor 488, goat anti-mouse secondary antibody AlexaFluor 546 and, respectively, goat anti-rabbit secondary antibody AlexaFluor 647, according to the manufacturer’s indications. Cell nuclei were stained using Hoechst 33,258 solution. The images were visualized and captured using the Nikon A1/A1R Confocal Laser Microscope System confocal microscope and corresponding software (Nikon Instruments Inc., version 5.11 64-bit, Melville, NY, USA). In addition, the quantification of the fluorescence levels was performed using ImageJ software (version 1.x bundled with 64-bit Java 8).

### 2.6. In Vivo Experiments

#### 2.6.1. Animals and in Vivo Experimental Protocol

Animals: The in vivo experimental protocol was approved by the Research Ethical Committee of Vasile Goldis Western University of Arad (approval no. 7/17.02.2020). The study complied with the Declaration of Helsinki and adhered to European and national regulations for laboratory animal research. Female BalbC mice, with seven mice per experimental group, were obtained from the Animal Facility of Vasile Goldis Western University of Arad and housed under conditions consistent with European and national standards.

Tumor induction: Breast cancer induction was achieved by injecting 106 4T1 murine mammary carcinoma cells, suspended in 200 µL of PBS, into the mammary gland. All injections were performed under anesthesia induced by intraperitoneal administration of ketamine (100 mg/kg body weight) and xylazine (10 mg/kg body weight). Two weeks after tumor cell injection, animals were evaluated clinically to detect the presence of neoformation tissue. Animals exhibiting tumors were selected for the next phase of the experiment, which involved the implantation of gelatin-based materials.

**Figure 2:**
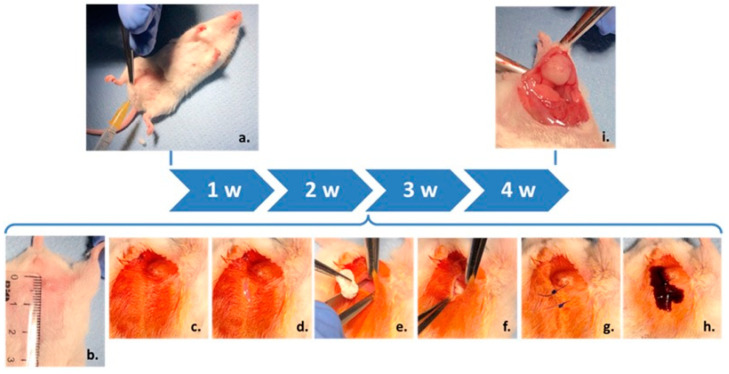
Schematic of the experiment and surgical procedure. (**a**) Injection of tumor cells into the mammary gland. (**b**) Visible tumor formation two weeks post-tumor cell injection. (**c**) Preoperative asepsis of the region of interest. (**d**) Skin incision, 1 cm in length, to create an approach path anterior to the tumor. (**e**) Separation of the skin and neoformation tissue from the muscular substrate to form the space required for implant placement, followed by positioning the test material dorsally to the neoformation tissue. (**f**) Approximation of wound edges. (**g**) Suturing of the skin incision. (**h**) Postoperative asepsis of the wound. (**i**) Collection of the biological samples.

Implantation: The experimental groups were structured as follows: control—healthy mice; tumor—mice with breast cancer without implants; 4T1 + GFAP—mice with breast cancer and GFAP implants; 4T1 + GFAP + PTX—mice with breast cancer and GFAP + PTX implants. The implants used in the study had a diameter of 0.5 cm. The abdominal area was shaved, disinfected with betadine, and a 1 cm incision was made anterior to the tumor to separate neoformation tissue from the underlying muscles, creating space for the implant. Materials were implanted, followed by closure with interrupted sutures and postoperative asepsis using betadine (Figure 2). Euthanasia was performed two weeks post-implantation under ketamine/xylazine anesthesia, and tumors with implanted materials, along with a 1 × 1 cm skin flap, were collected for histopathological and morphometric analyses.

#### 2.6.2. Gene Expression—Quantitative Real-Time PCR

The *Tp53*, *Nlrp3*, *caspase-1*, and *Il-1β* expression levels from the tissue samples were quantified by qRT-PCR. The total RNA was isolated using the classic protocol with TRIzol reagent. The concentration and purity of the obtained RNA samples were measured using a NanoDrop 8000 spectrophotometer. The cDNA synthesis was realized by employing the iScript cDNA Synthesis Kit and the Veriti 96-Well Thermal Cycler. Gene expression analyses were realized by employing a ViiA 7 Real-Time PCR System, Forget-Me-Not EvaGreen qPCR Master Mix, and specific primer sequences (Table 2). All of the samples were processed in triplicate, and the expression of mouse *GAPDH* was used as the reference. The expression levels were determined using the 2^−∆∆C(t)^ method, and the data were normalized to the control.

#### 2.6.3. Protein Expression—Western Blot Analysis

Western blot analysis was performed in order to evaluate the p53 and *caspase-1* protein expression levels from the tissue samples. Fragments of tissue were weighed and suspended in a ratio of 1:10 in lysis buffer (TrisHCl + 5 mM EDTA) and sonicated on ice using an UP50H Ultrasonic Processor (Hielscher Ultrasound Technology, Teltow, Germany) at an 80% amplitude, 1 cycle. After sonication, centrifugation of the samples was performed at 300 revolutions per minute (rpm), 10 min, 4 °C, and the total protein concentrations were measured by the Bradford method using 2 mg/mL BSA as the standard reagent. Samples were diluted in lysis buffer and loading buffer with 2-mercaptoethanol and denatured at 95 °C for 5 min. Subsequently, equal amounts of protein were separated with 12.5% polyacrylamide gels in TRIS-glycine-SDS buffer at 70 V for 30 min and 90 V for 90 min. Afterward, the proteins were transferred to a PVDF membrane in a wet transfer system, using a TRIS-glycine-methanol buffer, for 100 min. The detection of protein bands was performed using Western Breeze Chromogenic Kits. PVDF membranes were incubated with blocking solution for 30 min and incubated overnight with the primary antibodies p53 mouse monoclonal antibody (1:500) and *caspase-1* rabbit polyclonal antibody (1:1000). The membranes were incubated for 1 h with alkaline phosphatase-conjugated anti-mouse and anti-rabbit secondary antibodies. The protein bands were detected using the BCIP/NBT chromogenic substrate and visualized using the ChemiDoc Imaging System (BioRad, Hercules, CA, USA). The *GAPDH* was used as a reference protein.

### 2.7. Statistical Analysis

All experiments were performed in triplicate, and the experimental data were expressed as the mean ± standard deviation using GraphPad Prism software, version 6 (GraphPad Software Inc., San Diego, CA, USA). For the swelling tests, statistical significance was assessed using the one-way ANOVA method and Tukey algorithm to compare between groups. Values were considered significant for *p* < 0.0001. For the other characterizations, statistical significance was assessed using the one-way ANOVA method and Bonferroni algorithm to compare between groups. Values were considered significant for *p* < 0.05.

## 3. Results

### 3.1. Preparation and Characterization of PTX:Cyclodextrin Inclusion Complexes

**Figure 3:**
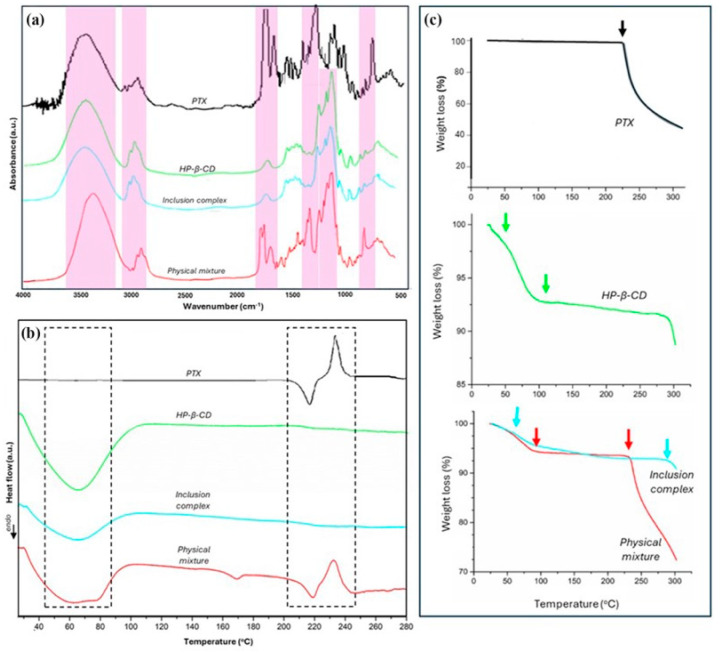
Evaluation of the inclusion complexes between HP-β-CD and PTX. (**a**) FTIR spectra, (**b**) DSC, and (**c**) TGA thermograms of pure PTX, HP-β-CD, PTX:HP-β-CD inclusion complexes, PTX and HP-β-CD physical mixture.

The FTIR test was the initial strategy employed to verify the successful encapsulation of PTX in the HP-β-CD derivative, with peak shifts or alterations in signal strength indicating structural modifications. Figure 3a displays the FTIR spectra obtained for the pure PTX, HP-β-CD, PTX:HP-β-CD inclusion complexes, and physical mixture of PTX with HP-β-CD. Pure PTX exhibited peaks at 3440 cm^−1^ for N-H stretching vibration and 2944 cm^−1^ for CH_2_ asymmetric and symmetric stretching vibrations. The signal at 1720 cm^−1^ corresponded to C=O stretching vibration from the ester group, while C-N stretching vibrations produced absorption bands at 1246 cm^−1^ [54,57,58]. The fingerprint section of the spectrum contained a peak at 710 cm^−1^, which indicates aromatic C-H out of plane deformation or C-C=O deformation [57,59]. The infrared spectrum of HP-β-CD revealed the presence of an O-H stretching vibration with strong absorption bands at 3416 cm^−1^, while C-H stretching vibrations generated peaks at 2928 cm^−1^. The primary absorption peaks at 1020, 1070, and 1150 cm^−1^, were assigned to the coupled C-C/C-O stretching vibrations and the antisymmetric stretching vibration of the C-O-C glycosidic bridge in the HP-β-CD molecules [60,61,62]. The spectrum for the PTX:HP-β-CD inclusion complexes closely resembled that of the pure HP-β-CD derivative, exhibiting no extra bands or alterations in band appearance, probably due to the total encapsulation of the drug within the cavities of the cyclodextrins. Nevertheless, modifications were observed within the spectrum of the physical mixture of PTX with HP-β-CD. Additional vibrational bands at 1736 cm^−1^ and 1704 cm^−1^, belonging to the C=O group, and at 1252 cm^−1^, corresponding to the C-N group, respectively, suggest the presence of free non-entrapped drug [54,63].

In addition, the formation of the inclusion complex was characterized by the DSC and TGA scans (Figure 3b,c). The DSC thermograms depicted in Figure 2b illustrate the behavior of the pure PTX, HP-β-CD, PTX:HP-β-CD inclusion complexes, and physical mixture of PTX with HP-β-CD. PTX exhibited an endothermic transition at 220 °C, indicative of the drug’s melting point and its crystalline structure. An exothermic transition indicating PTX breakdown was observable at ~240 °C [64]. An endothermic peak in the range of 40–90 °C, which indicates water desorption processes, could be observed in the DSC scans for HP-β-CD, the PTX:HP-β-CD inclusion complexes, and physical mixture of PTX with HP-β-CD. Supplementary thermal transitions did not appear in the pure HP-β-CD and PTX:HP-β-CD inclusion complexes, indicating that the PTX remained unmelted, safeguarded within the cyclodextrin cavity [31,54]. Conversely, the physical mixture presented slightly shifted PTX-specific endothermic and exothermic transitions (~218 °C and 235 °C, respectively) due to the melting and drug degradation.

TGA measurements (Figure 3c) were further used to verify the thermal stability of the PTX:HP-β-CD inclusion complexes and physical mixture of PTX with HP-β-CD in contrast to their pure components (PTX and HP-β-CD, respectively). The PTX’s mass began to drop (~2%) at around 240 °C; an accelerated thermal degradation process could be further observed, mainly due to the instability of the drug molecules [31]. Weight loss of HP-β-CD occurred in two stages: the first mass loss of ~7% at 100 °C can be attributed to the evaporation of unbound and constitutional moisture, and the second mass loss of ~3% in the temperature range of 100–300 °C indicating the molecules’ decomposition [65]. The TGA curve for the PTX:HP-β-CD inclusion complexes closely resembled that of HP-β-CD, exhibiting two primary mass losses with a remaining mass residue of ~90% at 300 °C. In contrast, the physical mixture exhibited a greater mass loss starting at around 250 °C, similar to the pure PTX, with a significant weight loss (~72%) at 300 °C. These findings suggest that the formation of the PTX:HP-β-CD inclusion complexes protects the PTX thermal degradation, whereas in the physical mixture, the degradation of the drug commenced earlier due to its superficial incorporation into the mixture [66].

### 3.2. Structural Characterization of Drug-Loaded Scaffolds

**Figure 4:**
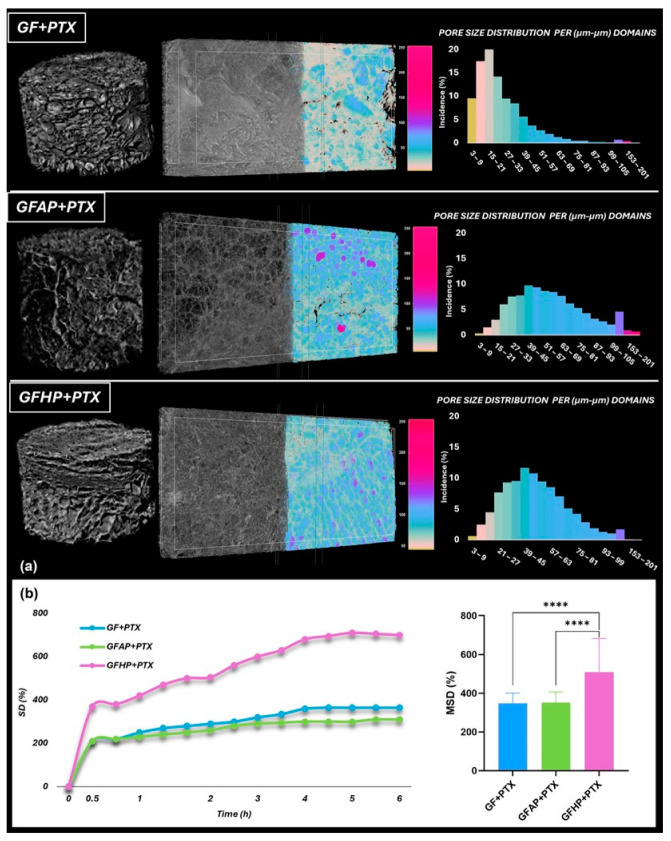
Structural characterization of the PTX-enriched scaffolds. (**a**) MicroCT pictures revealing the transversal internal microstructure of the scaffolds together with the tomograms (grayscale half) overlaid with CTAN-processed identified by color dataset and the charts of the quantitative assessment of pore diameter; (**b**) water uptake kinetics (left) and the equilibrium degree of swelling at 24 h (right). MSD (%) graph shows the mean ± standard deviation. (****) designates a significant difference (*p* < 0.0001).

Micro-CT analysis facilitated the assessment of the scaffolds’ morphology, particularly for studying porosity and pore systems. Figure 4a illustrates the reassembled tomograms of the specimens (left), where the transversal tomograms overlapped the color-coded dataset featuring pore dimensions (middle) and the plots of the quantitative analyses of pore size within the scanned samples (right). The freeze-drying of the synthetized samples generated highly porous structures with irregularly shaped pores, characterized by varying the structure thickness distribution and distinct pore size distribution relative to the solid content [67]. The GF + PTX samples exhibited a compact structure characterized by highly concentrated small pores, the majority of which had diameters ranging from 10 to 30 µm [68]. The incorporation of pectin (AP or HP) resulted in a notable increase in the pores’ size, facilitating the development of thinner and more flexible walls. The GFAP + PTX scaffolds exhibited smooth lamellar structures, thin walls, and pore sizes ranging from 30 to 70 µm whereas the presence of HP facilitated the development of interconnected, open pores with dimensions distributed in a Gaussian pattern ranging from 20 to 65 µm.

The water uptake profile of the PTX-containing hydrogels was further evaluated to verify the impact of the internal microstructure, porosity, and composition of the samples upon the swelling characteristics (Figure 4b). All of the specimens exhibited significant water absorption during the initial hour due to both the highly hydrophilic constituents of the scaffolds and their internal porosity. An equilibrium swelling ratio for the GF + PTX and GFAP + PTX scaffolds occurred after about 1 h of immersion, also proving comparable responses concerning the swelling performance after 24 h (MSD~350%) [69,70]. The experimental results obtained from the swelling measurements of GFHP + PTX showed that a linear swelling tendency was observed after about 4 h; moreover, a markedly improved water absorption capacity was observed at 24 h (MSD~700%). Such increased swelling could be attributed to a low cross-link density of the GFHP networks. The results showed that the GelMA and AP formulations were more likely to make dual-crosslinked networks formed through photopolymerization and the use of transglutaminase. The applied dual crosslinking strategy improved the stiffness of GFAP + PTX specimens, resulting in a decreased swelling. Surprisingly, the presence of AP did not appear to affect the swelling performance in comparison with the pectin-free scaffolds (GF + PTX).

### 3.3. In Vitro Biocompatibility Evaluation of 3D Biosystems

**Figure 5:**
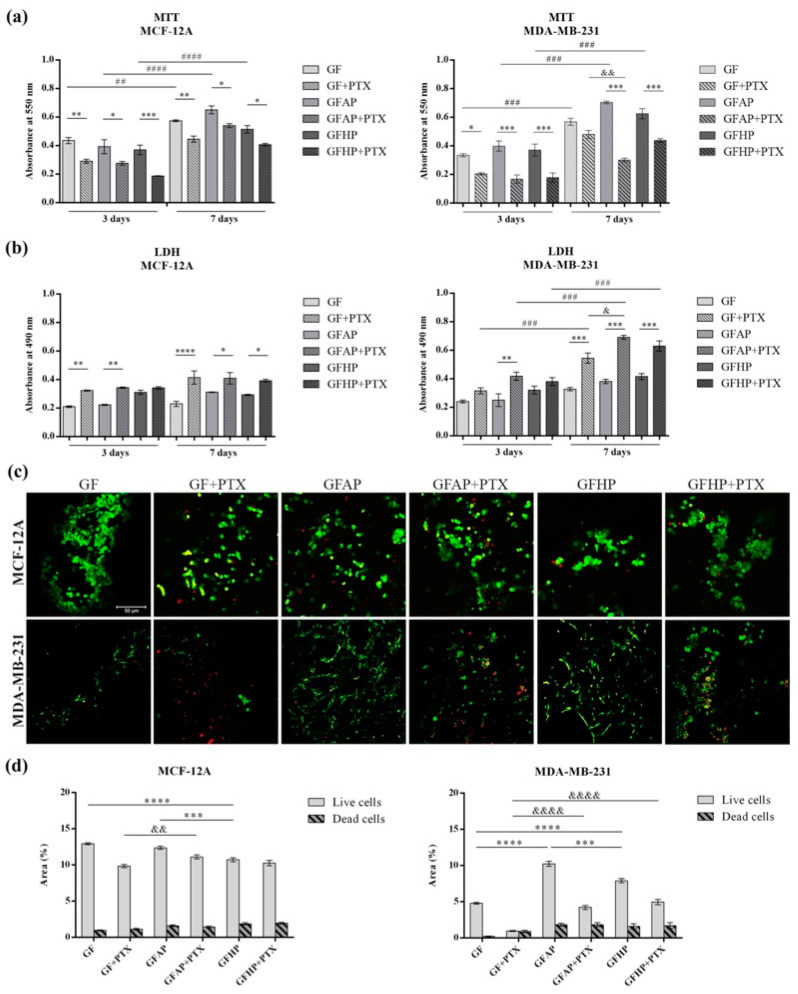
Biocompatibility assessment of the GF-based scaffolds in contact with MCF-12A and MDA-MB-231 breast cells. (**a**) Cell viability and proliferation profiles obtained after 3 and 7 days of incubation in standard conditions by the MTT assay. Statistical significance: * *p* < 0.05, ** *p* < 0.01, *** *p* < 0.001, ## *p* < 0.01, ### *p* < 0.001, #### *p* < 0.0001, and && *p* < 0.01. (**b**) Cytotoxicity evaluation of the proposed scaffolds after 3 and 7 days of incubation in standard conditions by the LDH assay. Statistical significance: * *p* < 0.05, ** *p* < 0.01, *** *p* < 0.001, **** *p* < 0.0001, ### *p* < 0.001, and & *p* < 0.05. (**c**) Qualitative evaluation of the live cells (green fluorescence) and dead cell nuclei (red fluorescence) in contact with the GF-based scaffolds. Scale bar: 50 μm. (**d**) Quantification of the green fluorescence and red fluorescence levels in all tested scaffolds. Statistical significance: *** *p* < 0.001, **** *p* < 0.0001, && *p* < 0.01, and &&&& *p* < 0.0001.

The MTT assay allowed for the quantification of the quality of the cell culture in contact with GF-based scaffolds enriched with PTX (Figure 5a). The results obtained after 3 days post-seeding indicated an overall high viability of MCF-12A cells in contact with the GF, GFAP, and GFHP scaffolds, without significant differences between these compositions. The presence of PTX in the composition of the scaffolds determined the decrease in the normal cell viability, the most significant decrease being observed in contact with the GFHP + PTX scaffold compared with GFHP (*p* < 0.001). Seven days post-seeding, the viability of the MCF-12A cells in contact with the PTX-enriched scaffolds indicated similar decreasing profiles compared with the non-enriched scaffolds. At the same time, the results obtained indicate the ability of the non-enriched scaffolds to support the proliferation of MCF-12A cells, with significant increases noted between 3 and 7 days of culture.

After 3 days of culture, the results of the MTT assay for the MDA-MB-231 cell line indicated a high viability in contact with the non-enriched scaffolds. However, the addition of PTX into the GF-based scaffolds determined a statistically significant decrease in the tumor cells’ viability, the most considerable decreases being observed in contact with GF + PTX compared with GF (*p* < 0.05), GFAP + PTX compared with GFAP (*p* < 0.001), and GFHP + PTX compared with GFHP (*p* < 0.001). After 7 days of seeding, the GFAP scaffold supported the highest level of cell viability when compared with GF and GFHP. A statistically lower cell viability profile was determined by GFAP + PTX compared with GFAP (*p* < 0.001) and GFHP + PTX compared with GFHP (*p* < 0.001). Moreover, GFAP + PTX promoted PTX activity and determined a considerable inhibition of the viability of tumor cells when compared with GF + PTX (*p* < 0.01). Additionally, the cells proliferated significantly from 3 to 7 days of in vitro cell culture on the GF-based scaffolds (*p* < 0.001).

In accordance with the MTT assay results, the LDH results indicated that the GF-based scaffolds induced certain levels of cytotoxicity upon the MCF-12A and MDA-MB-231 cells, which were significantly higher in contact with the PTX-enriched scaffolds (Figure 5b). After 3 days, statistical differences in the levels of LDH released by the MCF-12A cells were determined by the PTX-enriched GF (*p* < 0.05) and GFAP scaffolds (*p* < 0.05) compared with the GF and GFAP scaffolds. After 7 days, the highest cytotoxicity was promoted by GF + PTX compared with GF (*p* < 0.0001).

Regarding the MDA-MB-231 cells, the highest cytotoxicity registered after 3 days post-seeding was sustained by GFAP + PTX compared with GFAP (*p* < 0.01). After 7 days of culture, all PTX-enriched scaffolds sustained statistically significant higher cytotoxicity levels compared with the non-enriched scaffolds (*p* < 0.001). Additionally, the GFAP + PTX scaffold determined a statistically significantly higher cytotoxicity compared with GF + PTX (*p* < 0.05), suggesting the ability of GFAP to deliver the anti-tumor drug with higher efficiency. Between 3 and 7 days of culture, all GF-based scaffolds promoted the release and activity of PTX, as indicated by the statistically increased LDH levels (*p* < 0.001).

LIVE/DEAD fluorescent staining allowed for the assessment of the MCF-12A and MDA-MB-231 cell viability in contact with the GF-based scaffolds (Figure 5c). The images obtained indicated the formation of normal cell groups in contact with the GF-based scaffolds, suggesting the beneficial effects of the natural polymers from their composition. Small amounts of dead cell nuclei were identified in contact with the PTX-enriched scaffolds. A similar behavior was observed for the MDA-MB-231 tumor cells in contact with the proposed scaffolds. The GFAP and GFHP scaffolds promoted the elongated shape of tumor cells compared with the GF scaffold, suggesting the high biocompatibility of natural polymer-based scaffolds and the benefits of functionalizing the scaffolds’ composition. On the other hand, the presence of PTX determined the round phenotype of the MDA-MB-231 cells and promoted cell death, the most noticeable results being obtained in contact with the GFAP + PTX and GFHP + PTX scaffolds. In addition, quantification of the fluorescence levels was performed using ImageJ software, and the results suggested the high biocompatibility of the GF-based scaffolds and the cytotoxic effect of PTX (Figure 5d).

The results of the bio-mechanical characterization experiments allowed for the selection of 3D scaffolds. Therefore, the GF, GF + PTX, GFAP and GFAP + PTX scaffolds were used to evaluate the involvement of the *NLRP3* inflammasome activity in the TNBC treatment.

### 3.4. The Evaluation of Caspase-1 Activity, IL-1β Production, and ROS Generation

**Figure 6:**
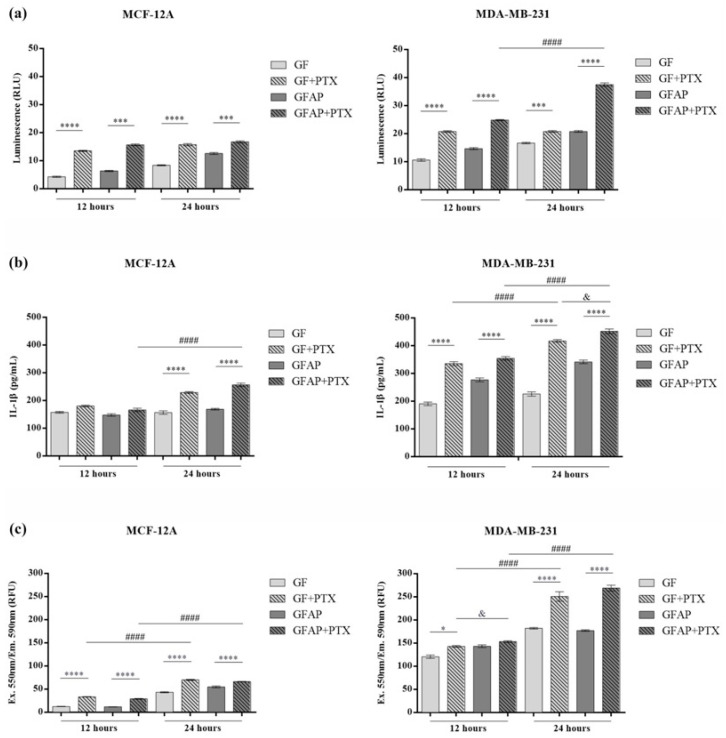
(**a**) Induction of *caspase-1* in breast cells in response to the composition of the GF-based scaffolds. *Caspase-1* activity in the MCF-12A cells and MDA-MB-231 cells in contact with the GF-based scaffolds after 12 h and 24 h by the Caspase-Glo 1 Inflammasome Assay. Statistical significance: *** *p* < 0.001, **** *p* < 0.0001, and #### *p* < 0.0001. (**b**) Secretion of IL-1β by breast cells in response to the composition of the GF-based scaffolds. The levels of IL-1β released into the culture media by the MCF-12A cells and MDA-MB-231 cells in contact with the GF-based scaffolds after 12 h and 24 h by the Lumit Human IL-1β Immunoassay. Statistical significance: **** *p* < 0.0001, #### *p* < 0.0001, and & *p* < 0.05. (**c**) Detection of extracellular ROS. H_2_O_2_ generation by the MCF-12A cells and MDA-MB-231 cells in contact with the GF-based scaffolds after 12 h and 24 h by the Amplex Red Hydrogen Peroxide/Peroxidase Assay. Statistical significance: * *p* < 0.05, **** *p* < 0.0001, #### *p* < 0.0001, and & *p* < 0.05.

The levels of *caspase-1* released into the culture media from the MCF-12A and MDA-MB-231 cells in contact with the GF-based scaffolds was assessed using the Caspase-Glo 1 Inflammasome Assay (Figure 6a). After 12 h of incubation of the normal cells, no notable differences in *caspase-1* activity were registered between the non-enriched scaffolds. However, the addition of PTX determined the statistical significance of *caspase-1* activity in contact with GF + PTX and GFAP + PTX compared with GF (*p* < 0.0001) and GFAP (*p* < 0.001), respectively. Similar profiles of *caspase-1* activity were obtained 24 h post-seeding, with the GF + PTX scaffold displaying the highest statistically significant increase compared with the GF control (*p* < 0.0001).

Regarding the MDA-MB-231 cells, the *caspase-1* levels were noticeably higher compared with the normal cells, suggesting the inflammatory potential of tumor cells. After 12 h post-seeding, statistically increased levels of *caspase-1* were observed in contact with GF + PTX compared with GF (*p* < 0.0001) and GFAP + PTX compared with GFAP (*p* < 0.0001), suggesting the anti-tumor and pro-inflammatory properties of PTX in contact with metabolically active tumor cells. After 24 h, similar profiles of *caspase-1* activity were observed between the non-enriched and PTX-enriched scaffolds, but the most significant increase was registered in contact with GFAP + PTX compared with GFAP (*p* < 0.0001). *Caspase-1* activity significantly increased between 12 h and 24 h in the MDA-MB-231 cells in contact with the GFAP + PTX scaffold (*p* < 0.0001), indicating the ability of PTX embedded in this composite to initiate pyroptosis as a consequence of *NLRP3* inflammasome activation.

Pyroptotic cells release pro-inflammatory mediators into culture media. Therefore, the Lumit Human IL-1β Immunoassay was used to determine the concentration of IL-1β released into the culture media by normal and tumor cells in contact with the proposed GF-based scaffolds (Figure 6b). After 12 h, the slightly increased profiles of IL-1β suggested the cytotoxic effect of PTX embedded into the scaffolds’ composition on normal breast cells. However, after 24 h, high levels of IL-1β released by normal cells were registered in contact with GF + PTX and GFAP + PTX compared with GF (*p* < 0.0001) and GFAP (*p* < 0.0001), respectively. A similar ascending profile of IL-1β was observed in contact with GFAP + PTX (*p* < 0.0001) between 12 h and 24 h of incubation in standard conditions.

IL-1β is secreted as a response to multiple pro-inflammatory stimuli including chemotherapy [71]. After 12 h, MDA-MB-231 cells exposed to PTX released statistically high levels of IL-1β compared with the corresponding control (*p* < 0.0001). Similar ascending profiles were noticed after 24 h of incubation in contact with GF + PTX compared with GF (*p* < 0.0001) and GFAP + PTX compared with GFAP (*p* < 0.0001). Moreover, a statistically significant difference was observed between these two compositions, with GFAP + PTX favoring the production of IL-1β compared with GF + PTX (*p* < 0.05). Stimulation of IL-1β secretion by GF + PTX (*p* < 0.0001) and GFAP + PTX (*p* < 0.0001) between 12 h and 24 h of incubation suggests the consequences of the prolonged exposure of tumor cells to the chemotherapeutic agent.

The levels of extracellular ROS released by the MCF-12A and MDA-MB-231 cells in contact with the GF-based scaffolds were measured using the Amplex Red Hydrogen Peroxide/Peroxidase Assay (Figure 6c). The obtained results indicated the increased concentration of H_2_O_2_ released into the culture media by normal and tumor cells in contact with the PTX-enriched scaffolds, starting from 12 h of incubation compared with the non-enriched scaffolds. After 24 h, the presence of PTX determined a significant (*p* < 0.0001) production of H_2_O_2_ by the normal cells.

In the case of the MDA-MB-231 tumor cells, the exposure to GF + PTX and GFAP + PTX led to a significant increase in the H_2_O_2_ concentration after 12 h of incubation. After 24 h, the MDA-MB-231 cells produced a twofold higher concentration of H_2_O_2_ compared with the first interval time, maintaining the same significantly increased profile in contact with the PTX-enriched scaffolds compared with the non-enriched scaffolds (*p* < 0.0001).

### 3.5. In Vitro Breast Cancer and NLRP3 Inflammasome-Associated Gene Expression Variation

**Figure 7:**
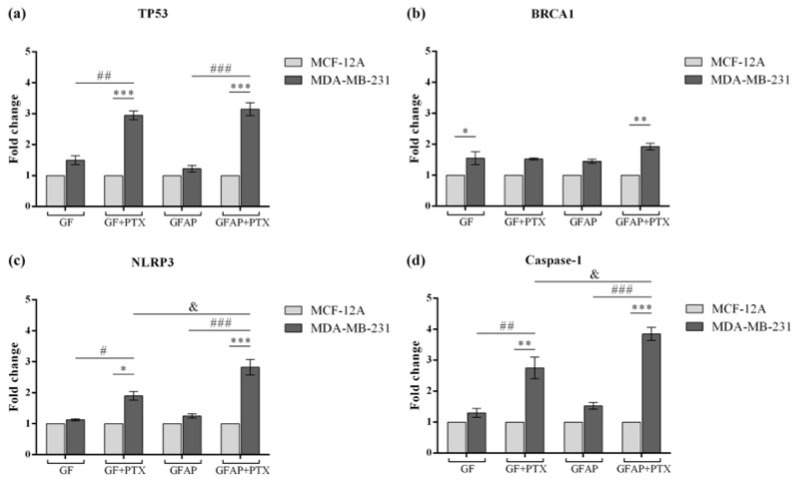
Evaluation of breast cancer and *NLRP3* inflammasome-associated genes’ expression. Differential (**a**) *TP53*, (**b**) *BRCA1*, (**c**) *NLRP3*, and (**d**) *caspase-1* gene expression in normal MCF-12A and tumor MDA-MB-231 cells exposed to GF-based scaffolds. Statistical significance: * *p* < 0.05, ** *p* < 0.01, *** *p* < 0.001, # *p* < 0.05, ## *p* < 0.01, ### *p* < 0.001, and & *p* < 0.05.

In order to understand the impact of the PTX-enriched GF-based scaffolds on breast cancer pathogenesis and *NLRP3* inflammasome mechanism, we performed qRT-PCR to investigate the variation in the expression of the *TP53*, *BRCA1*, *NLRP3*, and *caspase-1* genes in MDA-MB-231 tumor cells compared with normal cells (Figure 7). The contact of MDA-MB-231 cells with the GF-based scaffolds determined the increase in *TP53* expression, and the most significant increases were recorded for GF + PTX (*p* < 0.001) and GFAP + PTX (*p* < 0.001) compared with the expression of *TP53* in the control cells. Moreover, the addition of PTX into the composition of the GF and GFAP scaffolds determined a statistically significant upregulation of *TP53* (*p* < 0.01 and *p* < 0.001, respectively), suggesting the involvement of *TP53* in various cellular processes such as cell cycle or programmed cell death.

The *BRCA1* expression was also modified as a result of the breast tumor cells being in contact with the GF-based scaffolds compared with the normal cells. The composition of the GF scaffold determined a statistically significant increase in *BRCA1* expression (*p* < 0.05), while the compositions of GF + PTX and GFAP promoted slightly ascending, but statistically insignificant, *BRCA1* expression profiles. The most significant variation of *BRCA1* expression was obtained in contact with the GFAP + PTX scaffold (*p* < 0.01), suggesting that the composition of GFAP promotes PTX activity with the highest efficiency in breast tumor cells.

Regarding the expression of the inflammasome-associated genes, the GF + PTX scaffold promoted the statistically significant increase in *NLRP3* expression in the MDA-MB-231 cells compared with both the normal cells (*p* < 0.05) and GF composition (*p* < 0.05), suggesting the capacity of PTX to stimulate the inflammasome’s mechanism in tumor cells. Similarly, an even more significant increase in *NLRP3* expression was registered in contact with the GFAP + PTX scaffold compared with the normal cells (*p* < 0.001) and unenriched GFAP composition (*p* < 0.001). Furthermore, a significant difference in *NLRP3* expression was observed in tumor cells exposed to the two PTX-containing scaffolds, the most suitable for PTX delivery being GFAP (*p* < 0.05).

*Caspase-1* is an effector of the *NLRP3* inflammasome and its expression was analyzed to investigate the pyroptosis process associated with this complex. As expected, *caspase-1* expression increased as a consequence of the tumor cells’ contact with the GF + PTX and GFAP + PTX scaffolds compared with the normal cells and unenriched scaffolds, GF and GFAP (*p* < 0.01 and *p* < 0.001, respectively). In accordance with the *NLRP3* expression variation, the GFAP + PTX scaffold promoted a significant increase in the *caspase-1* expression in tumor cells compared with the GF + PTX scaffold (*p* < 0.05), strengthening the results obtained previously.

### 3.6. In Vitro Breast Cancer and NLRP3 Inflammasome-Associated Protein Expression Variation

**Figure 8:**
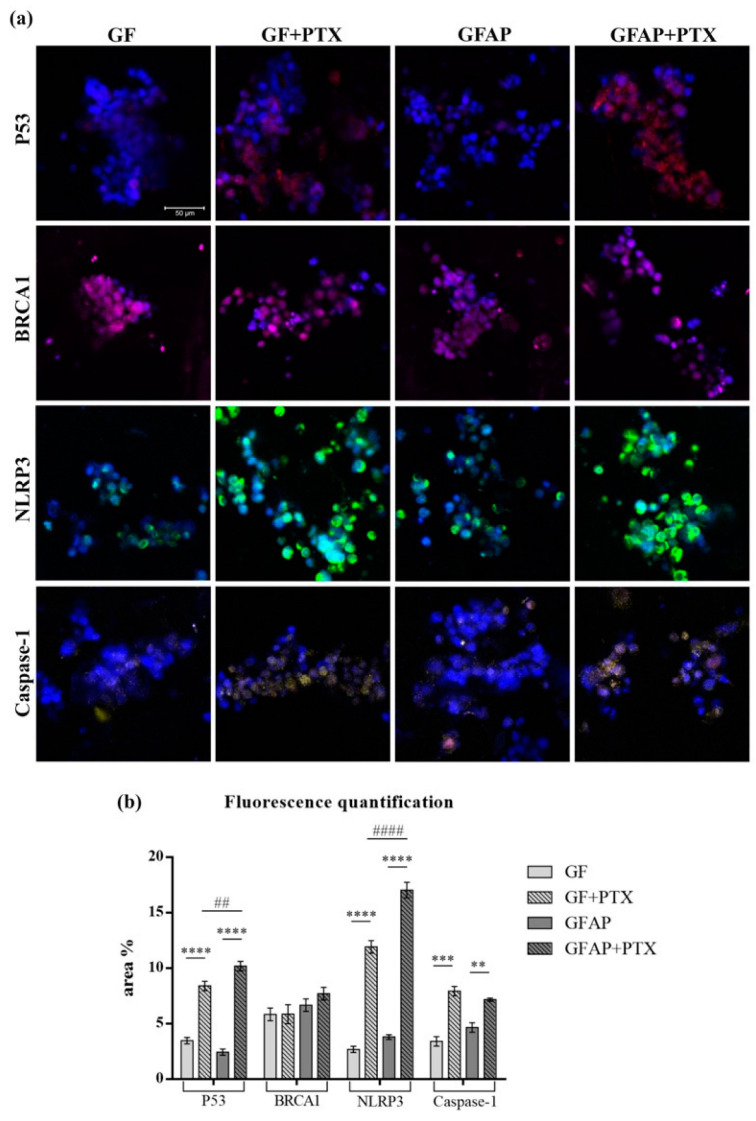
P53, *BRCA1*, *NLRP3*, and *caspase-1* protein expression in MDA-MB-231 cells seeded in GF-based scaffolds. (**a**) P53, *BRCA1*, *NLRP3*, and *caspase-1* protein expression variation in MDA-MB-231 cells seeded into GF-based scaffolds. P53 is shown in red, *BRCA1* is shown in violet, *NLRP3* is shown in green, *caspase-1* is shown in yellow, and cell nuclei are shown in blue. Scale bar 50 µm. (**b**) Quantification of fluorescence intensity. Statistical significance: ** *p* < 0.01, *** *p* < 0.001, **** *p* < 0.0001, ## *p* < 0.01, and #### *p* < 0.0001.

Scientific studies conducted in recent years have highlighted the impact of *NLRP3* inflammasome signaling on the breast cancer mechanism. The results obtained by confocal microscopy coupled with immunofluorescence confirmed the gene expression data (Figure 8).

The addition of PTX into the composition of the GF and GFAP scaffolds determined the notable increase in p53, *NLRP3*, and *caspase-1* protein expression in the MDA-MB-231 cells, suggesting the activation of pyroptosis cell death associated with *NLRP3* inflammasome assembly as a consequence of PTX treatment of breast tumor cells.

The quantification of red fluorescence associated with p53 protein expression indicated a statistically increased expression in contact with GF + PTX (*p* < 0.0001) and GFAP + PTX (*p* < 0.0001) in comparison with the corresponding GF and GFAP scaffolds. As expected, the p53-associated fluorescence was significantly higher in contact with GFAP + PTX compared with GF + PTX (*p* < 0.01). In the case of *BRCA1*, the associated violet fluorescence maintained relatively constant in contact with all GF-based scaffolds, with a statistically insignificant increase in contact with GFAP + PTX. The statistically increased profile of green fluorescence associated with *NLRP3* expression and yellow fluorescence associated with *caspase-1* expression in MDA-MB-231 cells exposed to GF + PTX (*p* < 0.0001 and *p* < 0.001, respectively) and GFAP + PTX (*p* < 0.0001 and *p* < 0.01, respectively), compared with the unenriched scaffolds suggested the activation of the *NLRP3* inflammasome and pyroptosis.

### 3.7. In Vivo Breast Cancer and NLRP3 Inflammasome-Associated Markers’ Expression Variation

**Figure 9:**
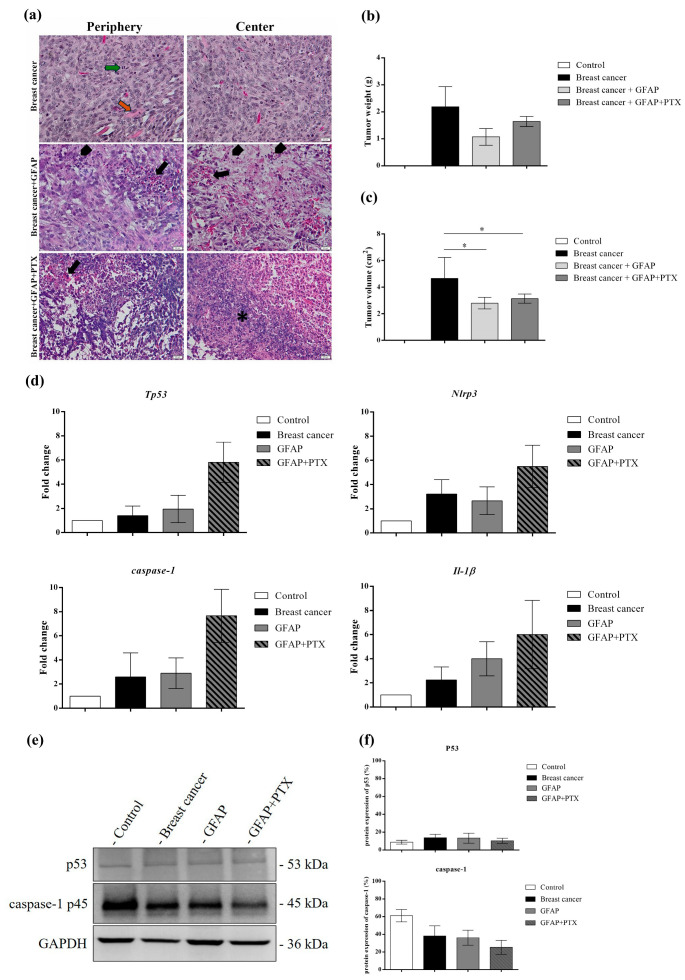
In vivo investigation of *NLRP3*-associated TNBC. (**a**) The histological appearance of the breast tumors after the implantation of GFAP and GFAP + PTX compared with the control tumor; mitosis (green arrow), muscle cell (orange arrow), inflammatory in-filtrate (black arrow); * necrosis; col. H&E. Scale bar 20 μm. (**b**) Tumor weight (g). (**c**) Tumor dimension (cm^2^). Statistical significance: * *p* < 0.05. (**d**) Differential expression of *Tp53*, *Nlrp3*, *caspase-1*, and *Il-1β* genes measured by qRT-PCR analysis. (**e**) Western blot protein evaluation of p53 and *caspase-1* after GFAP and GFAP + PTX scaffolds were implanted at the tumor site. (**f**) Quantification of blot images. Data were normalized to *GAPDH* and expressed as percentages related to the control.

In female Balb/c mice injected with 4T1 tumor cells, histopathological analysis at 4 weeks revealed malignant anaplastic tumors composed of undifferentiated cells, distinct from the original parenchyma. The rapid proliferation of cancer cells in the mammary parenchyma and stroma was accompanied by pronounced morphological alterations and heightened mitotic activity. Local invasion into muscle fibers further highlighted the tumor’s aggressive phenotype (Figure 9a). These observations align with the known malignancy and invasiveness of 4T1 cells, widely recognized as a reliable model for evaluating anti-tumor drug efficacy due to their resemblance to human metastatic breast cancer. In the group receiving the peritumoral implantation of GFAP, inflammatory infiltrates appeared within the tumors by the second week, accompanied by tumor cells exhibiting condensed, pyknotic nuclei. Necrotic tissue, characterized by disorganized cellular debris, indistinct cell boundaries, and eosinophilic cytoplasm, was also observed. In contrast, the combination of GFAP and PTX exhibited enhanced anti-tumor activity. This was evidenced by extensive tumor lysis at the periphery, an increased prevalence of tumor cells with pyknotic nuclei, and a more prominent central necrotic zone containing inflammatory cells and cellular debris. Morphometric measurements confirmed these findings, demonstrating a significant reduction in tumor size (Figure 9b) and volume (Figure 9c).

The following experiments included in the study were intended to validate the in vitro results. Therefore, the expression levels of the *Tp53, Nlrp3*, *caspase-1*, and *Il-1β* genes from the mice tumor samples were analyzed by employing qRT-PCR (Figure 9d). The obtained results suggested a notable increase in *Tp53* expression in the GFAP + PTX group compared with mice in which a breast tumor was induced, suggesting the role of upregulated *Tp53* in cell cycle regulation and programmed cell death initiation [72]. Similar ascending profiles were registered for the expression of the *NLRP3* inflammasome components, such as *Nlrp3*, *caspase-1*, and *Il-1β*, in the presence of the GFAP + PTX implant compared with the positive control group, suggesting the efficacy of PTX embedded into GFAP scaffolds to initiate pyroptosis tumor cell death as a consequence of *NLRP3* inflammasome activation. As expected, the unenriched GFAP scaffolds did not stimulate a considerable variation in gene expression compared with the positive control group, indicating the benefits of natural origin scaffolds on cellular behavior.

The in vivo effects of PTX-enriched scaffolds on TNBC-derived tumors were studied by analyzing the protein expression of p53 and *caspase-1* by Western blot (Figure 9e). Recent studies have indicated that p53-positive TNBC pathologies are more sensitive to PTX-based chemotherapy than p53-negative TNBC [73]. The obtained results indicated an insignificant variation in p53 expression between the breast cancer positive control group and GFAP implant group, while the presence of GFAP + PTX determined a notable variation of p53, suggesting the role of p53 as a tumor suppressor and mediator of programmed cell death [74]. Pyroptosis cell death is identified by active *caspase-1*, and the processing of pro-*caspase-1* into its p20 and p10 subunits is required for the initiation of this type of cell death. Pyroptosis initiation determines the release of active *caspase-1*, IL-1β, and IL-18 through GSDMD-derived pores. Therefore, the obtained results suggest the initiation of pro-*caspase-1*’s processing mechanism in the presence of the GFAP + PTX scaffold and the slight increasing variation of active *caspase-1* in the tissue lysate after the implant procedure.

## 4. Discussion

Breast cancer remains an unpredictable neoplasia and is associated with limited therapeutic alternatives and a high mortality rate. The frequent approach for breast cancer is surgical removal of the tumor, named mastectomy or lumpectomy, depending on the tumor size [75]. Subsequently, a method for reconstructing excised breast tissue is suggested in order to improve the oncology patients’ quality of life. Over the years, numerous approaches for immediate or delayed post-mastectomy breast reconstruction have been studied; most of the time, immediate reconstruction is chosen to reduce the number of surgical interventions and to improve the esthetic results. Prosthetic reconstruction is among the most commonly used post-mastectomy breast reconstruction techniques. Currently, special interest has been given to the tissue engineering field and to obtain scaffolds capable of supporting the regeneration of breast tissue [76]. An interesting study realized by Zhu et al. highlighted the capacity of foamed GelMA to promote the vascularization and adipogenic differentiation of the grafts and as a carrier for 3D adipose-derived stem cell spheroids for breast defect repair after mastectomy. The obtained results indicated the versatility of the synthesis method and the scaffolds’ porous structure, which facilitated the cells’ viability, proliferation, and differentiation [77].

Scaffolds based on natural polymers, such as collagen, cellulose, gelatin, alginate, or pectin, are characterized by biodegradable hydrophilic structures, high porosity, and good biocompatibility, also being indicated for the controlled release of anti-tumor agents (e.g., PTX, 5-fluorouracil, cisplatin, doxorubicin, etc.) in the case of a residual tumor microenvironment and the prevention of recurrence [78,79]. The controlled release of chemotherapeutic drugs at the tumor site is an important aspect for tumor eradication, and to ensure their controlled delivery, various platforms have been developed such as scaffolds, liposomes, or nanoparticles, etc. [80,81].

For example, Liu et al. investigated the capacity of hyaluronic acid-based micelles to deliver PTX and to inhibit breast tumor growth both in vitro and in vivo. The obtained results indicated that free PTX inhibited the growth of the tumor and normal cells, and the proposed PTX-loaded micelles determined the inhibition of the tumor cells while maintaining the viability of normal cells. Furthermore, the results suggested that free PTX induced toxic side effects, significant inflammation, and necrosis in the inflamed tissue due to non-targeting. On the other hand, the hyaluronic acid-based micelles discharged PTX within the tumor microenvironment and maintained stability in normal physiological conditions [82]. Another study realized by Yang et al. illustrated the efficiency of PTX and gemcitabine enriched-hyaluronic acid and 6-(2-nitroimidazole)-hexylamine polymers to accumulate at tumor sites, enter into tumor cells via endocytosis, and initiate MDA-MB-231 cell death. Similar to the previous results, the researchers observed that free drugs exhibited toxic side effects, while their encapsulation stimulated the targeted anti-tumor effect, in a time-dependent manner [83].

Moreover, the breast tumor microenvironment is associated with *NLRP3* inflammasome activation, characterized by pyroptosis cell death and the release of pro-inflammatory cytokines. The scientific literature suggests the interconnection between *NLRP3* inflammasome signaling and breast cancer, especially TNBC progression [4]. For example, Gomez et al. indicated that different dimensions (12–200 nm) of silica nanoparticles exhibited immunomodulatory effects by inducing the secretion of interleukins via *NLRP3* inflammasome activation, in a dose-dependent manner [84]. Consequently, there is great interest in the identification of an efficient and biocompatible anti-tumor platform that is capable of targeted drug delivery while supporting breast tissue reconstruction. In this context, we proposed original 3D scaffolds based on GelMA and pectin loaded with PTX–cyclodextrin inclusion complexes as anti-tumor platforms.

The results of the biocompatibility and cytotoxicity assays indicated the overall good biocompatibility of the unenriched scaffolds (GF, GFAP, and GFHP) in contact with both the normal MCF-12A and tumor MDA-MB-231 cells. The addition of PTX determined a substantial decrease in the tumor cell viability, demonstrating PTX’s effectiveness as an anti-tumor agent. Another study conducted by Zhou et al. illustrated the efficiency of double-modified nanoparticles based on gelatin and bovine serum albumin in delivering chemotherapy to breast cancerous regions. The results proved that PTX-loaded nanoparticles have the ability to inhibit the growth of tumor cells and induce apoptosis [85].

The presence of PTX stimulated the mechanism of the *NLRP3* inflammasome and pyroptosis in MDA-MB-23 cells, as indicated by the increased activity of *caspase-1* and high concentration of IL-1β released into the cell culture media. Numerous studies have reported the capacity of anti-tumor drugs to stimulate the *caspase-1*-dependent cell death of tumor cells; however, the role of the *NLRP3* inflammasome and pyroptosis in the breast cancer context is still ambiguous and requires in-depth molecular studies for a full understanding. For example, a recent study investigated the ability of cellulose nanofibers and pectin-based 3D hydrogels enriched with 5-fluorouracil to suppress the growth of breast cancer cells and activate *NLRP3*-associated pyroptosis. The obtained results highlighted the good biocompatibility and capacity of the proposed 3D hydrogels to facilitate 5-fluorouracil’s therapeutic efficacy and to implicitly stimulate *caspase-1* activity in breast tumor cells [79].

Regarding *caspase-1* expression, our results were consistent with previous studies. However, *caspase-1* cleavage involves an elaborate mechanism that is not fully understood yet. Among the first publications regarding the cleavage process of *caspase-1* was the one realized by Broz et al., which suggested the existence of unprocessed and fully processed *caspase-1*. The experimental data indicated that pyroptosis can be initiated by activated pro-*caspase-1*, and that active *caspase-1* is rapidly released from the cells after pyroptosis initiation, except for those retained by ASC [86,87].

The results obtained in this study indicated a time-dependent increase in ROS generation in breast tumor cells exposed to PTX-containing scaffolds, the highest increase being recorded for the GFAP + PTX scaffold, suggesting the ability of this composition to sustain the PTX effect. However, the generation of ROS stimulated by PTX anti-tumor activity suggests that oxidative stress may be responsible for the development of side effects associated with chemotherapy. Over the years, the association between PTX and ROS generation was intensely investigated, and the results indicated the involvement of multiple mechanisms [88,89].

Even though the results of in vitro studies are valuable, advanced in vivo studies are also needed to better understand the mechanisms of the *NLRP3* inflammasome and pyroptosis in the breast cancer context. Therefore, the in vivo results obtained in this study contribute to the progress of the oncology field. The results of our in vitro and in vivo studies are consistent with experimental data from the scientific literature, highlighting the importance of identifying efficient carriers than can selectively induce tumor cell death.

## 5. Conclusions

In this study, we evaluated novel 3D scaffolds based on GelMA and two distinct types of pectin enriched with PTX–cyclodextrin inclusion complexes for their ability to inhibit the growth of breast tumor cells and stimulate *NLRP3*-associated pyroptosis. The physico-chemical characterization of the scaffolds indicated an optimal porosity for supporting breast tissue reconstruction. The scaffolds’ composition facilitated normal breast cells’ viability and proliferation, while the presence of PTX into the scaffolds’ compositions stimulated *caspase-1* activity, IL-1β secretion, and ROS production by the breast tumor cells. Moreover, in vivo investigations suggested the activation of *NLRP3* inflammasome and pyroptosis mechanisms as a consequence of PTX delivery by the GF-based scaffolds at the tumor sites. The obtained results contribute to the understanding of the *NLRP3* inflammasome pathway in the context of breast pathologies and approve the ability of PTX-enriched GF-based scaffolds to counteract the growth of breast tumor cells by inducing *NLRP3* inflammasome activation and pyroptosis cell death, acting as potential therapeutic scaffolds for breast cancer therapy.

## Figures and Tables

**Table 1:** Primer sequences.

Gene	Nucleotide Sequence			
human *GAPDH*	F	GTCTCCTCTGACTTCAACAGCC	R	ACCACCCTGTTGCTGTAGCCAA
human *TP53*	F	CGAGATGTTCCGAGAGCTGAAT	R	TTTATGGCGGGAGGTAGACTGA
human *BRCA1*	F	GCTTGACACAGGTTTGGAGTATGC	R	GAGAGTTGGACACTGAGACTGGTT
human *NLRP3*	F	GCACGTGTTTCGAATCCCAC	R	CCTGCTGGCTCCGGTGCTCC
human *caspase-1*	F	GCCTGTTCCTGTGATGTGGAG	R	TGCCCACAGACATTCATACAGTTTC

**Table 2:** Primer sequences.

Gene	Nucleotide Sequence			
mouse *Gapdh*	F	CATCACTGCCACCCAGAAGACTG	R	ATGCCAGTGAGCTTCCCGTTCAG
mouse *Tp53*	F	CAGTCTGGGACAGCCAAGTC	R	CTTCTGTACGGCGGTCTCTC
mouse *Nlrp3*	F	TCACAACTCGCCCAAGGAGGAA	R	AAGAGACCACGGCAGAAGCTAG
mouse *caspase-1*	F	GGCACATTTCCAGGACTGACTG	R	GCAAGACGTGTACGAGTGGTTG
mouse *Il-1β*	F	TGGACCTTCCAGGATGAGGACA	R	GTTCATCTCGGAGCCTGTAGTG

## Data Availability

The data presented in this study are available on request from the corresponding author. The data are not publicly available due to privacy.
